# Interleukin-1β Promoter Polymorphism Enhances the Risk of Sleep Disturbance in Alzheimer’s Disease

**DOI:** 10.1371/journal.pone.0149945

**Published:** 2016-03-03

**Authors:** You Yin, Yan Liu, Xiao Pan, Rui Chen, Peng Li, Hui-Juan Wu, Zheng-Qing Zhao, Yan-Peng Li, Liu-Qing Huang, Jian-Hua Zhuang, Zhong-Xin Zhao

**Affiliations:** 1 Department of Neurology, Changzheng Hospital, Second Military Medical University, Shanghai, China; 2 Institute of Neuroscience and MOE Key Laboratory of Molecular Neurobiology, Neuroscience Research Center of Changzheng Hospital, The Second Military Medical University Shanghai, Shanghai, China; 3 Department of Pharmacy, Xinhua Hospital Affiliated to Shanghai Jiaotong University School of Medicine, Shanghai, China; Duke University, UNITED STATES

## Abstract

Sleep alleviates Alzheimer’s disease (AD)-related neuropathological processes, whereas sleep disturbance in AD patients is associated with elevated peripheral inflammatory cytokine levels. In the present study, we assessed interleukin (IL)-1β and APOEε4 polymorphisms for association with susceptibility of sleep disturbances in AD patients. A total of 123 pretreated AD patients and 120 age-, gender- and education level-matched healthy controls were recruited for two consecutive full-night polysomnography and measurement of Epworth Sleepiness Scale (ESS) scores for sleep-wake disturbance. Their genomic DNA was analyzed for IL-1β and APOEε4 SNPs using ligase detection reaction (LDR) technology. Blood levels of IL-1β, IL-6, and tumor necrosis factor alpha (TNF-α) were measured using ELISA after lipopolysaccharide (LPS) stimulation. The odds ratio and 95% confidence interval for genotype-specific risk were calculated using an unconditional logistic regression model and adjusted by age, gender, educational levels, body mass index (BMI), and activities of daily living (ADL). Compared to the non-APOEε4/ε4 genotype, APOEε4/ε4 significantly increased the risk of AD (APOEε4/ε4 vs. non-APOEε4/ε4, adjusted OR = 4.33, 95% CI = 1.33–14.10, *p* = 0.015). Compared to the IL-1β CC genotype (-31), the TT genotype significantly increased the risk of AD (TT vs. CC, adjusted OR = 1.72, 95% CI = 1.13–2.61, *p* = 0.010). AD patients carrying the APOEε4 allele and the IL-1β TT genotype showed less time in bed, longer sleep latency and REM latency, more awakenings, and a lower SWS percentage than those carrying CC/CT combined genotypes. In addition, blood IL-1β levels were significantly greater in AD patients carrying both the APOEε4 allele and the IL-1β-31TT genotype than in those carrying the APOEε4 allele and the -31 TC or CC genotype. In conclusion, this study provides the first evidence indicating that the IL-1β-31TT genotype and homozygous APOEε4 combined are associated with increased risk of developing AD with sleep disturbance.

## Introduction

Sleep disturbances or disorders represent a medical condition that is sometimes serious enough to disturb normal physical, social, mental, and emotional functions and have been thought to be associated with many progressive dementing diseases [1–3]. A cross-sectional clinic- and community-based survey has demonstrated that approximately 40% of patients with Alzheimer’s disease (AD) experience sleep disturbances [4]. AD is a degenerative disease and the most common form of dementia, with memory loss as an early symptom. To date, the cause and pathogenesis of AD and the association of AD with sleep disturbance are not fully understood [5]. Our previous studies have shown that sleep disturbance in late-onset AD is associated with cognitive and functional decline and that daytime sleepiness in mild and moderate AD patients is associated with elevated serum levels of proinflammatory factors [6]. However, it remains to be determined how sleep disturbance occurs in AD patients.

To date, a number of studies have demonstrated that the inflammatory process not only contributes to AD [7–10], but also is involved in the regulation of physiological sleep [11–14]. For example, the proinflammatory cytokine interleukin (IL)-1β is a main component in inflammatory pathways and is overexpressed in the brain of AD patients [15]. An animal study showed that when IL-1β was infused into the lateral cerebral ventricles of rabbits, IL-1β induced a dose-dependent increase in slow-wave sleep [11], suggesting a sleep-promoting effects of IL-1. Another study has shown that IL-1β causes a dose-dependent increase in non-rapid eye movement (NREM) sleep but suppresses rapid eye movement (REM) sleep in mice; however, IL-1 type I receptor knockout mice did not respond to IL-1 treatment [12]. Mechanistically, IL-1β directly alters discharge patterns of neurons in the hypothalamic and brainstem circuits and regulates sleep-wake behavior [13]. In addition, IL-1 promotes NREM sleep and induces sleep loss-associated symptoms, including sleepiness, fatigue, and poor cognition [14]. IL-1β has been reported to be a “master regulator” in brain inflammation, and its signaling is necessary for expression of other cytokines such as IL-6 and tumor necrosis factor alpha (TNF-α) [15].

In this regard, several studies have shown associations between IL-1 polymorphisms and AD development. IL-1 includes a family of three closely related proteins, i.e., IL-1α, IL-1β, and IL-1 receptor antagonist (IL-1RA) [16]. IL-1 is localized at chromosome 2q13-21 [17]. Single nucleotide polymorphisms (SNPs) in the IL-1β gene promoter region (-511 and -31) alter the inducing effect of lipopolysaccharide (LPS) on IL-1 gene transcription, leading to susceptibility to inflammatory diseases [18–20]. Previous studies have also demonstrated that IL-1β-511 and -31 SNPs are associated with AD risk [21–24], while the IL-1β-1473 SNP affects triglyceride and IL-6 metabolism [25]. Moreover, to date, there is evidence showing that SNPs of APP, PSEN1 and PSEN2 are associated with familial AD, and the epsilon-4 allele of APOE is associated with late-onset AD [26–28]. In this study, we tested our hypothesis that IL-1β SNPs together with APOEε4 SNPs can contribute to sleep disturbances in AD patients and the expression of proinflammatory factors (IL-1β, IL-6, and TNF-α). We assessed IL-1β (-511C>T, -31 T>C and -1473 C>G) and APOEε4 SNPs in pretreated AD patients and healthy controls for their risk of developing sleep disturbances in late-onset AD.

## Materials and Methods

### Study subjects

In this study, we recruited 123 newly diagnosed and drug-free probable AD patients (45 males, 78 females) From aged between 65 and 89 years old (mean: 72 ± 7 years) from the Dementia Center, Chang Zheng Hospital, the Second Military Medical University (Shanghai, China) between January 2010 and December 2013. The diagnosis of dementia was confirmed by the investigators at Chang Zheng Hospital based on the Diagnostic and Statistical Manual of Mental Disorders (DSM-IV) criteria, and probable AD patients were selected according to the NINCDS-ADRDA criteria [29]. During the same time period, 120 age-, gender-, and education level-matched elderly healthy individuals who were receiving routing physical examinations at Chang Zheng Hospital were also recruited to this study. Both cases and controls were Han Chinese who lived in the Shanghai district. Subjects with inflammatory diseases were excluded from the study. This study was approved by the Ethics Committee of the Second Military Medical University of China. All AD patients had a moderate (10–18 points) or mild (19–24 points) Mini Mental State Examination (MMSE) score. All AD patients and/or their caretakers provided written informed consent to participate in this study. All healthy controls also provided written informed consent.

Patient evaluations included medical history, physical and neurological examinations, chest radiography, electroencephalography (EEG), and nuclear magnetic resonance imaging (MRI) of the brain. Laboratory blood tests were performed to exclude metabolic causes of dementia (FT3, FT4, TSH, vitamin B12, and folic acid) and to assess the inflammatory status (erythrocyte sedimentation rate and C-reactive protein) of each recruited subject. Only subjects showing a normal erythrocyte sedimentation rate and C-reactive protein values were selected for the study. Furthermore, all patients received neuropsychological tests including the Hamilton Anxiety Scale, Hamilton Depressive Scale, and the Mini Mental State Examination (MMSE), and functional autonomy evaluation using the Activities of Daily Living (ADL) scale. Sleep disturbance was evaluated with the Pittsburgh Sleep Quality Index (PSQI) and Epworth Sleepiness Scale (ESS) based on information provided by the caregivers during the interview.

All subjects had no concomitant inflammatory, autoimmune, neurological, infectious, or psychiatric disease, and were not taking antiplatelet therapy, antibiotics, nonsteroidal anti-inflammatory drugs, neuropsychiatric drugs, acetylcholinesterase (AchE) inhibitors, or antineoplastic, corticosteroid, or immunosuppressive drug treatments in the preceding one month. In addition, all the patients had an erythrocyte sedimentation rate (ESR) within reference levels. Subjects with obstructive sleep apnea syndrome or Sundowning syndrome were excluded from this study.

### Evaluation of sleep disturbance

Polysomnography (PSG) was used to evaluate sleep disturbance. Specifically, both patients and healthy controls underwent two consecutive nocturnal polygraphic sleep recordings (the first used for adaptation), according to a recent study [30]. PSG was performed between 8:00 p.m. (lights out) and 7:30 a.m. according to personal habits in all subjects. Polygraphic sleep recording included two EEGs (C4-A1, C3-A2), two electrooculograms, submental electromyograms, ECG, nasal-oral flow (thermistor), thoracic and abdominal strain gauges, finger pulse oxymetry, digital microphone recording, and electromyography of the bilateral tibialis anterior muscles. Conventional sleep variables were evaluated according to standard criteria [31]. In addition, apnea/hyperpnoea episodes and periodic leg movements were scored against conventional criteria.

### Genotyping

Genomic DNA was extracted from 3 mL of EDTA-preserved peripheral blood using a QIAamp DNA Blood Midi Kit (Qiagen, Hilden, Germany), according to the manufacturer’s instructions. IL-1β rs1143623/-1473, rs16944/-511, and rs1143627/-31 and ApoE rs429358 and rs7412 SNPs were genotyped by Shanghai Generay Biotech Co., Ltd. (Shanghai, China) using the Multiplex polymerase chain reaction-ligase detection reaction (PCR-LDR) method. Specifically, the primers for amplification of IL-1β rs1143623 were 5'-AAATCAGAAGGCTGCTTGGA-3' (forward) and 5'-ATGGGTGAATGGGAATTTGA-3′ (reverse); for IL-1β rs16944, 5'-TGGCTAGGGTAACAGCACCT-3' (forward) and 5'-GGGTACAATGAAGGGCCAAT-3' (reverse); and for IL-1β rs1143627, 5'-CCCTTCCATGAACCAGAGAA-3' (forward) and 5'-GCTGAAGAGAATCCCAGAGC -3' (reverse). The primers for amplification of ApoE rs429358 and rs7412 SNPs were 5'-AGGGTGCTGATGGACGAGACCATGAAGGAG-3' (forward) and 5'-GCTCACGGATGGCGCTGAGGCCGCGCTCGG-3' (reverse). The PCR amplification was carried out in a total volume of 20 μL that included 50 ng of genomic DNA, 2 μL of PCR buffer, 0.6 μl of 3 mM Mg^2+^, 2 μL of 2 mM dNTP, and 0.3 U of Taq DNA polymerase (TaKaRa, Otsu, Japan). The conditions were as follows: an initial at 95°C for 15 min and 35 cycles of 94°C for 30 s, 56°C for 60 s, and 72°C for 1 min, and a final extension at 72°C for 7 min.

Next, we synthesized three probes for LDR for each SNP locus, which included two specific probes and one common probe. The specific probes used to discriminate the specific bases were 5'-TTTTTTTTTTTTTTTTCGCGGTACTGCACCAGGCGGCCGCA-3' and 5'- TTTTTTTTTTTTTTTTTTCGCGGTACTGCACCAGGCGGCCGCG-3' for ApoE rs429358 SNP, 5'-TTTTTTTTTTTTTTTTTTCCCCGGCCTGGTACACTGCCAGGCG-3' and 5'-TTTTTTTTTTTTTTTTTTTTCCCCGGCCTGGTACACTGCCAGGCA-3' for ApoE rs7412 SNP, 5'-TTTTTTTTTTTTTTTTTTTTGGTATCGTGCTCGCTCTGCATTATC-3' and 5'- TTTTTTTTTTTTTTTTTTTTTTGGTATCGTGCTCGCTCTGCATTATG-3' for IL-1β rs1143623 SNP, 5'-TTTTTTTTTTTTTTTTTTTTTTACCTTGGGTGCTGTTCTCTGCCTCG-3' and 5'-TTTTTTTTTTTTTTTTTTTTTTTTACCTTGGGTGCTGTTCTCTGCCTCA-3' for IL-1 rs16944 SNP, and 5'-TTTTTTTTTTTTTTTTTTTTTTTTCAGTTTCTCCCTCGCTGTTTTTATG-3' and 5'-TTTTTTTTTTTTTTTTTTTTTTTTTTCAGTTTCTCCCTCGCTGTTTTTATA-3' for IL-1β rs1143627 SNP. The common probe was phosphorylated at the 5'-end and labeled at the 3'-end with 6-carboxyfluorescein (FAM). For rs429358, rs7412, rs1143623, rs16944, and rs1143627 SNPs, the common probes were 5'-P-CACGTCCTCCATGTCCGCGCCCAGCTTTTTTTTTTTTTTTT-FAM-3', 5'-P-CTTCTGCAGGTCATCGGCATCGCGGTTTTTTTTTTTTTTTTTT-FAM-3', 5'-P-CAAGGGAGTGAGCCTCTGTGCAAGTTTTTTTTTTTTTTTTTTTTT-FAM-3', 5'-P-GGA(GA)CTCTCTGTCAATTGCAGGAGCTTTTTTTTTTTTTTTTTTTTTT-FAM-3', and 5'-P-GCTTTCAAAAGCAGAAGTAGGAGGCTTTTTTTTTTTTTTTTTTTTTTTT-FAM-3', respectively. LDRs were performed in a 10-μL reaction volume containing 1 μL of PCR product, 1 μL of Taq DNA ligase buffer, 1 μl of each probe (12.5 pmol), and 0.1 U of Taq DNA ligase (New England Biolabs, Ipswich, MA, USA) at 95°C for 2 min, 35 cycles of 94°C for 30 s, and 50°C for 2 min. Next, 1 μl of LDR product was subjected to DNA sequencing using an ABI 3730XL DNA sequencer. In addition, representative PCR products were also subjected to direct DNA sequencing to confirm the accuracy of this method, and the data showed 100% concordant results.

### Detection of plasma cytokine levels

Whole blood samples (3 mL) were collected between 8:00 am and 9:00 am following the first sleep recording for the LPS-stimulated whole-blood assay as described in a previous study [19]. The aliquots of whole blood were mixed 1:1 with RPMI 1640 culture medium and incubated with 100 ng/mL LPS (Escherichia coli O26:B6; Difco Laboratories, Detroit, MI, USA) at 37°C for 4 h. Next, the plasma was separated from the whole blood for detection of IL-1β, IL-6, and TNF-α levels using ELISA kits (R&D Systems, Minneapolis, MN, USA), according to the manufacturer’s protocols. Each plate included a standard curve and known positive/negative controls. The absorbance (A) rate was read against the blank at 450 nm using a microtiter ELISA reader (BioRad, Hercules, CA, USA). Intra- and inter-assay coefficients of variations were less than 10% for all analyses. The assay sensitivity for IL-1β was <2 pg/mL, for IL-6 it was <8 pg/mL, and for TNF-α it was 0.5–5.5 pg/mL.

### Statistical analysis

Statistical analyses were performed using the SPSS v.16.0 software package (SPSS, Chicago, IL, USA). Hardy-Weinberg equilibrium was evaluated using the χ^2^ test. Genotype and allelic frequencies were calculated by direct counting of the genotypes and alleles, and simple proportion calculation. Allele frequencies were calculated by allele counting as previously described [32]. Comparison of the genotype distribution in patients and healthy controls were performed by means of two-sided contingency tables using the χ^2^ test. The odds ratio (OR) and 95% confidence interval (CI) for genotype-specific risk were calculated using an unconditional logistic regression model and adjusted by age, gender, educational levels, body mass index (BMI) and activities of daily living (ADL). *p*<0.05 was considered statistically significant in this study.

## Results

### Demographic data

There was no significant difference in age, gender, or educational level between cases and controls (Table 1). However, body mass index and ADL levels were significantly different between the AD patients and controls (Table 1).

**Table 1:** Comparison of Demographic and Control Measurements between Cases and Controls.

	Controls	AD patients	*p*
**Age (yrs.)**	70.9±7.3	72.1±7.2	0.198
**Gender (M/F)**	41/79	45/78	0.693
**Education (yrs.)**	11.6±2.8	11.4±3.3	0.611
**BMI**	24.3±2.9	22.6±2.8	<0.001
**ADL**	14.0±0.0	17.1±5.9	<0.001
**MMSE**	28.3±1.5	19.7±5.4	<0.001

BMI, body mass index; ADL, activities of daily living score; MMSE, mini mental state examination score.

### Association of APOE, IL-1β rs1143623 (-1473), rs16944 (-511), and rs1143627 (-31) SNPs with risk of sleep disturbances in AD patients

The genotype distributions of APOE rs429358 and rs7412 and IL-1β rs1143623 (-1473), rs16944 (-511), and rs1143627 (-31) SNPs in AD patients and controls were consistent with the Hardy-Weinberg equilibrium. The frequencies of the -31TT genotype and homozygous APOEε4/ε4 in AD patients were different from those of the healthy controls. Compared to the non-APOEε4/ε4 genotype, APOEε4/ε4 significantly increased the risk of AD (APOEε4/ε4 vs. non-APOEε4/ε4, adjusted OR = 4.33, 95% CI = 1.33–14.10, *p* = 0.015). Compared to the IL-1β CC genotype (-31), the TT genotype significantly increased the risk of AD (TT vs. CC, adjusted OR = 1.72, 95% CI = 1.13–2.61, *p* = 0.010). The findings suggest that the IL-1β-31TT genotype and homozygous APOEε4 are associated with the risk of developing AD with sleep disturbance. There was no statistically significant difference in the distribution frequencies of IL-1β-1473 and -511 SNPs between AD patients and controls (Table 2).

**Table 2:** Association of IL-1β-1473/-511/-31 and ApoEε4 SNPs with AD.

Locus	Genotypes and alleles	AD (n = 123) n(%)	Controls (n = 120)n(%)	OR (95% CI)^a^	*P*
ApoE	Absence of ε4	80 (65.0)	87 (72.5)	1.00	
	Presence of ε4	43 (35.0)	33 (27.5)	1.61 (0.89–2.90)	0.113
	No ε4/ε4	108 (87.8)	116 (96.7)	1.00	
	ε4/ε4	15 (12.2)	4 (3.3)	4.33 (1.33–14.10)	0.015
IL-1β					
rs1143623 (-1473)	GG	24 (19.5)	33 (27.5)	1.00	
**G/C (G>C)**	GC	64 (52.0)	60 (50.0)	1.46 (0.74–2.88)	0.280
	CC	35 (28.5)	27 (22.5)	1.35 (0.89–2.06)	0.158
	GC/CC	99 (80.5)	87 (72.5)	1.50 (0.79–2.85)	0.216
	C allele	0.545	0.475		
rs16944 (-511)	TT	18 (14.6)	21 (17.5)	1.00	
**T/C(T>C)**	TC	60 (48.8)	57 (47.5)	1.377 (0.63–3.00)	0.422
	CC	45 (36.6)	42 (35.0)	1.240 (0.82–1.88)	0.305
	TC/CC	105 (85.4)	99 (82.5)	1.493 (0.73–3.06)	0.274
	C allele	0.610	0.588		
rs1143627 (-31)	CC	15 (12.2)	29 (24.2)	1.00	
**C/T(C>T)**	CT	57 (46.3)	60 (50.0)	1.64 (0.77–3.53)	0.203
	TT	51 (41.5)	31 (25.8)	1.72 (1.13–2.61)	0.010
	CT/TT	36 (87.8)	91 (75.8)	2.03 (0.98–4.20)	0.056
	T allele	0.646	0.508		

^a^Adjusted for age, gender, and educational level.

### Differential nocturnal polysomnography and sleep scale results in AD patients carrying the APOEε4 allele and the IL-1β TT genotype at -31

As shown in Table 3, AD patients carrying the IL-1β TT genotype at the -31 site had the lowest MMSE score (18.6 ± 5.7), highest ESS score (9.4 ± 6.6), longest REM latency (168.2 ± 33.0 min), and lowest percentages of REM (16.6 ± 5.3%) and SWS (0.2 ± 0.2%) among the three IL-1β-31 genotypes; in contrast, no significant difference was found among the IL-1β-31 genotypes in the healthy controls. There was no significant difference in the respiratory disturbance index (RDI) among the genotypes of IL-1β-31 SNPs in either the AD patients or the healthy controls (Table 3). As shown in Table 4, the incidence of daytime sleepiness (ESS >10) was significantly greater in AD patients with the IL-1β TT genotype than in those with the CT or CC genotype (58.8% vs. 47.4% and 20.0%, *p* = 0.029) and greater in patients with the T allele than in those with the C allele (54.7% vs. 37.9, *p* = 0.012). As shown in Table 5, AD patients carrying the APOEε4 allele and the IL-1β TT genotype showed less time in bed (506.7 ± 18.3 min); longer sleep latency (24.2 ± 16.2 min) and REM latency (152.8 ± 27.5 min); more awakenings (18.6 ± 5.1); and a lower SWS percentage (0.1 ± 0.2%) than those carrying CC/CT combined genotypes.

**Table 3:** Sleep measurements between AD patients and controls with different genotypes of IL-1β-31 SNPs.

	Controls (n = 120)	Control-CC (n = 29)/AD-CC(n = 15)	CT(n = 60)/ CT (n = 57)	TT (n = 31)/TT (n = 51)
**MMS**	28.3 ± 1.5	28.8±1.5	28.2±1.4	28.1±1.5
		23.6 ± 3.6^#^	19.6 ± 5.2^#^^^^	18.6 ± 5.7^#^^^^
**ESS scale**	4.7 ± 3.1	4.3±3.2	5.2±3.3	4.2±2.5
		6.0 ± 4.1	7.4 ± 4.6^#^	9.4 ± 6.6^#^^†^
**Time in bed (min)**	571.2 ± 48.4	570.8±39.9	567.8±51.6	578.1±49.8
		531.9 ± 61.5^#^	539.5 ± 54.4^#^	536.4 ± 50.4^#^
**Total sleep time (min)**	365.6 ± 124.1	369.1±100.5	364.0±125.2	365.4±144.3
		321.4 ± 79.4*	361.1 ± 106.1^†^	319.1 ± 49.4^#^
**Sleep latency (min)**	9.8 ± 5.4	11.5±5.2	9.5±5.5	8.8±5.3
		19.3 ± 13.0^#^	17.9 ± 14.8^#^	18.8 ± 14.1^#^
**Sleep efficiency (%)**	66.9 ± 20.2	70.2±18.2	67.5±20.0	62.6±22.3
		67.1 ± 17.5	67.7 ± 16.0	60.1 ± 10.9*
**Awakenings (n)**	13.6 ± 5.0	12.4±5.1	13.6±4.4	14.9±5.8
		15.5 ± 4.9	14.9 ± 4.1	18.6 ± 6.2^#^
**WASO (min)**	169.5 ± 131.7	145.0±119.7	163.7±130.1	203.4±142.3
		155.3 ± 107.6	156.3 ± 87.3	207.3 ± 76.0*
**REM latency (min)**	127.7 ± 40.0	127.6±34.1	125.0±39.3	132.8±46.5
		122.4 ± 50.3	129.6 ± 56.6	168.2 ± 33.0#^
**REM (%)**	16.3 ± 4.2	17.6±3.9	16.2±4.1	15.2±4.3
		19.9 ± 5.2^#^	17.4 ± 6.3	16.6 ± 5.3^
**Stage1 (%)**	27.3 ± 9.5	26.2±9.2	26.4±10.0	29.9±8.7
		26.1 ± 16.0	24.5 ± 10.4	26.9 ± 20.7
**Stage2 (%)**	54.8 ± 7.8	53.8±7.8	55.8±7.7	54.0±7.9
		53.4 ± 12.4	57.8 ± 9.8	57.6 ± 19.3
**SWS (%)**	1.9 ± 2.9	2.3±3.2	1.8±2.7	1.8±2.9
		1.6 ± 1.0	1.2 ± 0.7	0.2 ± 0.2#†
**RDI**	3.7 ± 1.8	3.3±1.8	3.8±1.8	3.8±1.9
		3.7 ± 1.0	3.6 ± 1.7	3.6 ± 1.4
**PLMSI**	3.7 ± 2.3	3.7±2.1	3.6±2.3	3.9±2.7
		2.0 ± 1.4#	2.7 ± 2.5#	3.1 ± 1.9

PLMSI, periodic limb movements of sleep index; RDI, respiratory disturbance index

*P<0.05 compared with controls

#P<0.01 compared with controls

†P<0.05 compared with CC (-31)

^P<0.01 compared with CC (-31).

**Table 4:** Association of the IL-1β (-31) SNP with AD patients with/without daytime sleepiness.

AD (-31) n (%)	C allele	T allele	*P*	CC	CT	TT	*P*
ESS>10	33 (37.9)	87 (54.7)	0.012	3 (20.0)	27 (47.4)	30 (58.8)	0.029
ESS<10	54 (62.1)	72 (45.3)		12 (80.0)	30 (52.6)	21 (41.2)	

**Table 5:** Sleep measurements in AD patients with different genotypes of IL-1β (-31) SNPs.

	AD- CC/CT (n = 53)	AD- TT (n = 27)	*P*	AD+ CC (n = 3)	AD+ CT (n = 16)	AD+TT (n = 24)	*P*
ESS	7.2 ± 4.3	8.3 ± 7.4	0.393	3.33 ± 0.6	7.8 ± 5.2	10.5 ± 5.5*	0.051
Time in bed (min)	534.6 ± 55.7	506.7 ± 18.3	0.014	597.3 ± 57.6	537.7 ± 51.6	569.7 ± 54.3	0.093
Total sleep time (min)	343.7 ± 100.6	317.8 ± 35.3	0.199	411.0 ± 136.7	371.2 ± 101.1	320.1 ± 62.4	0.074
Sleep latency (min)	16.8 ± 14.4	24.2 ± 16.2	0.040	14.7 ± 8.5	23.6 ± 14.5	12.7 ± 7.7	0.011
Sleep efficiency (%)	66.0 ± 17.4	62.8 ± 7.3	0.365	74.7 ± 16.7	71.6 ± 11.0	57.1 ± 13.4*	0.002
Awakenings (n)	15.0 ± 4.5	18.6 ± 5.1	0.002	13.3 ± 4.0	15.3 ± 3.7	18.8 ± 7.4	0.12
WASO	167.9 ± 98.1	180.1 ± 40.7	0.516	131.2 ± 108.8	121.6 ± 48.7	237.2 ± 94.5*	0.000
REM latency (min)	122.2 ± 56.4	152.8 ± 27.5	0.010	120.3 ± 51.8	149.2 ± 48.6	185.4 ± 30.4#	0.004
REM (%)	18.9 ± 6.5	17.3 ± 5.4	0.286	16.9 ± 5.4	14.9 ± 3.8	15.8 ± 5.2	0.74
Stage 1 (%)	24.5 ± 11.2	26.0 ± 20.3	0.674	21.8 ± 13.0	26.6 ± 13.6	28.0 ± 21.6	0.85
Stage 2 (%)	56.3 ± 9.4	58.6 ± 19.9	0.486	60.5 ± 7.9	58.3 ± 13.9	56.5 ± 18.9	0.89
SWS (%)	0.4 ± 0.8	0.1 ± 0.2	0.023	0.8 ± 0.7	0.2 ± 0.4#	0.2 ± 0.2#	0.008

AD- means AD patients without ApoEε4 (n = 80), and AD+ means AD patients with ApoEε4 (n = 43).

*p<0.05 compared with CC (-31) + APOEε4

^#^*p*<0.01 compared with CC (-31) + APOEε4.

### Levels of IL-1β, IL-6, and TNF-α in cell culture supernatant after LPS stimulation

**Fig 1:**
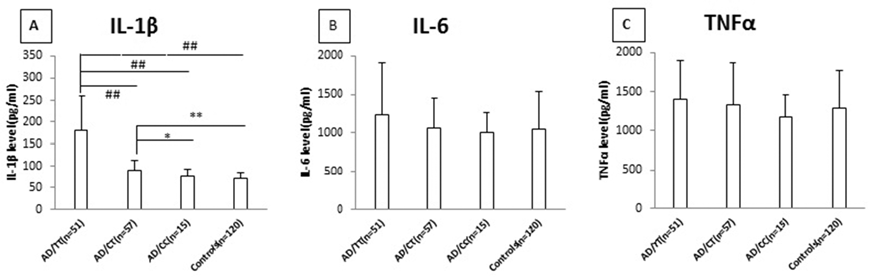
Levels of LPS-induced IL-1β, IL-6, and TNF-α in peripheral blood leukocytes of AD patients with different genotypes of -31 (IL-1 promoter) and healthy controls. ^##^*p*<0.01, comparison between the TT genotype and the CT genotype, the CC genotype, and the healthy controls. **p<0.01, comparison between the CT genotype and the healthy controls; *p = 0.030, comparison between the CT genotype and the CC genotype.

Fig 1 shows LPS-induced protein levels of IL-1β, IL-6, and TNF-α in subjects carrying different IL-1β-31 genotypes. Specifically, IL-1β levels were significantly greater in AD patients carrying the IL-1β-31TT genotype than in those carrying the CT or the CC genotype and the healthy controls (Fig 1*A*); in addition, a significant difference was observed between the CT or the CC genotype and the healthy controls (Fig 1A). No significant difference in the levels of IL-6 and TNF-α was found among the IL-1β-31 genotypes (Fig 1*B* and 1*C*).

**Fig 2:**
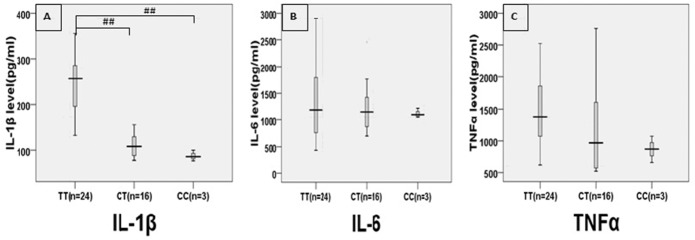
Levels of LPS-induced IL-1β, IL-6, and TNF-α production in peripheral blood leukocytes of AD patients with the APOE 4 allele and different genotypes of -31 (IL-1 promoter). ^##^*p*<0.001, comparison between the average levels of the TT genotype and the TC and the CC genotypes.

Fig 2 shows LPS-induced protein levels of IL-1β, IL-6, and TNF-α in AD patients carrying the APOEε4 allele and different IL-1β-31 genotypes. Specifically, IL-1β levels were significantly greater in AD patients carrying both the APOEε4 allele and the IL-1β-31TT genotype than in those carrying the APOEε4 allele and the -31 TC or CC genotype (Fig 2*A*). No significant difference in the levels of IL-6 and TNF-α was found among AD patients carrying the APOEε4 allele and different IL-1β-31 genotypes (Fig 2*B* and 2*C*).

## Discussion

Frequently, AD patients experience sleep disorders in the daytime and/or nighttime [4], and certain genetic variations could be responsible. In the present study, we associated IL-1β and APOEε4 SNPs with susceptibility to sleep disturbances in AD patients and found that AD patients carrying the -31TT genotype in the IL-1β promoter plus the APOEε4 allele were susceptible to sleep disturbance. Mechanistically, these two genotypes showed elevated peripheral inflammation levels of IL-1β after LPS stimulation. The synergistic effect of the IL-1β-31TT genotype and the APOEε4 allele led to an increased susceptibility of inducing IL-1β, IL-6 and TNF-α overexpression and sleep disturbance in AD patients. Thus, our data indicate that the IL-1β-31TT genotype and the APOEε4 allele were associated with sleep disturbances in AD patients and that these two SNPs synergistically enhanced LPS-induced IL-1β, IL-6 and TNF-α overexpression in white blood cells from AD patients with sleep disturbances.

Previous studies have demonstrated that altered expression of IL-1β in brain inflammation and IL-1β SNPs are associated with AD, but other studies have shown controversial data [33]. The present study has shown a positive association of IL-1β and APOEε4 SNPs with susceptibility to sleep disturbances in AD patients. Payão et al. [24] have shown that the IL-1β-31 polymorphism was associated with AD risk. Two other studies have revealed that IL-1β-31T is involved in inflammation-related lower cognition and is driven by a dementia process [33, 34]. However, the data from Ma et al. [35] did not show any associations between IL-1β-31T to C and late-onset AD in a Chinese population. Our data showed that there was no association of the IL-1β-511 T>C polymorphism with AD risk, which is consistent with the data from a large meta-analysis of 17 association studies between AD and the IL-1β-511 T>C polymorphism (including 3371 AD patients and 3338 controls) [16, 36], although four other studies of Caucasians showed a positive association between the -511TT genotype and AD (OR = 1.32) with a total of 1158 AD patients and 863 controls [21, 37–39]. These contradictory results could be explained by differences in the prevalence of some polymorphisms in different ethnic groups [40]. Furthermore, to the best of our knowledge, no studies have shown an association between -1473 (rs1143623) and AD risk. Our data showed that the association was still statistically significant when age, gender, and educational level were included in a multivariate regression model analysis.

Furthermore, using nocturnal PSG and sleep scale (ESS) data in the present study, we found a strong and consistent assocaition between the IL-1β-31 promoter TT genotype and sleep disturbances in the daytime (ESS score) and nighttime (REM latency, REM%, and SWS%) of AD patients compared to the healthy controls. Moreover, we found a synergistic effect of the IL-1β-31TT genotype and the APOEε4 allele on sleep-wake disturbance during the daytime (ESS) and sleep disturbances at nighttime (sleep efficiency%, WASO (min), REM latency, and REM%) in AD patients. To the best of our knowledge, no studies have attempted to examine the association of the IL-1β promoter -31 genotype and APOEε4 with sleep disturbances in AD patients. Because the APOEε4 allele has been identified as an independent risk factor for late-onset AD [26–28], our results suggest that the high incidence of sleep disturbances in AD patients has an inherent genetic basis.

However, the patient population with AD is heterogeneous and it is very common for patients with similar MMSE scores and clinical cognitive symptoms to show significant differences in sleep disturbances, cytokine production, and intensity of inflammation [6]. In the present study, we linked these parameters to genetic background variations and showed that the -31TT genotype of the IL-1β promoter confers significantly greater LPS-induced IL-1β expression in white blood cells from AD patients. These results are partly consistent with a previous study shwoing that LPS-induced IL-1β levels were significantly greater in trauma patients carrying the IL-1β TT genotype (at the -31 bp site) than in those with the TC and CC genotypes [41]. Another study has indicated that IL-1β may be a “master regulator” of brain inflammation [15]. Our findings are in agreement with this study. However, multiple genes and factors could be involved in the development of sleep disturbances and the expression of inflammation-related genes in AD patients. Previous studies have shown that APOE has an ability to regulate the expression of inflammation-related genes, including IL-1β production in vitro and in vivo [42, 43]. Our data showed a synergistic effect of the APOEε4 allele and the IL-1β-31TT genotype on LPS-induced high expression of IL-1β and sleep disturbances in AD patients. Although our results only showed the high expression of peripheral inflammatory cytokines, a peripheral immune alteration in AD patients may be triggered by brain immune activation through the inflammatory signaling pathway from the brain to the periphery [44, 45]. Peripheral immune alterations may be linked to progressive deposition of Abeta1-42 within the central nervous system [46]. Thus, we speculated that the sleep disturbance in AD patients may be associated with a central inflammatory reaction induced by a synergistic effect of -31TT (IL-1β promoter) and the APOEε4 allele.

There are some limitations to the study: (1) Only Han Chinese patients in Shanghai district were enrolled in this study, which minimized background noise for the study. However, although Chinese Han accounts for 90% of the population in China and 19% of global population [47], enrollment of a single ethnicity in this single-center study may limit the generalizability of our findings. (2) As a retrospective case-control study, this study was unable to elicit a causal relationship in the association of the combined IL-1β-31TT/ homozygous APOEε4 with AD with sleep disturbance. A future prospective cohort study is needed for this purpose. (3) The use of a subjective assessment of sleep-wake disturbance such as ESS could have resulted in some measurement error, although we collected information from the caregivers rather than from the AD patients to minimize the possibility.

## Conclusions

In conclusion, this study provides the first evidence indicating that the IL-1β-31TT genotype and homozygous APOEε4 combined are associated with increased risk of developing AD with sleep disturbance.
